# A Single-Turnover Kinetic Study of DNA Demethylation Catalyzed by Fe(II)/α-Ketoglutarate-Dependent Dioxygenase AlkB

**DOI:** 10.3390/molecules24244576

**Published:** 2019-12-13

**Authors:** Lyubov Yu. Kanazhevskaya, Irina V. Alekseeva, Olga S. Fedorova

**Affiliations:** Institute of Chemical Biology and Fundamental Medicine (ICBFM), 8 Lavrentiev Ave., Novosibirsk 630090, Russia

**Keywords:** dioxygenase AlkB, DNA demethylation, conformational dynamics, protein–DNA interactions, stopped-flow method, single-turnover kinetics, fluorescent probes, FRET analysis

## Abstract

AlkB is a Fe(II)/α-ketoglutarate-dependent dioxygenase that repairs some alkylated bases of DNA and RNA in *Escherichia coli*. In the course of catalysis, oxidation of a co-substrate (α-ketoglutarate, αKG) leads to the formation of a highly reactive ‘oxyferryl’ enzyme-bound intermediate, Fe(IV) = O, ensuring hydroxylation of the alkyl nucleobase adducts. Previous studies have revealed that AlkB is a flexible protein and can adopt different conformations during interactions with cofactors and DNA. To assess the conformational dynamics of the enzyme in complex with single- or double-stranded DNA in real-time mode, we employed the stopped-flow fluorescence method. N^1^-Methyladenine (m^1^A) introduced into a sequence of 15-mer oligonucleotides was chosen as the specific damage. Single-turnover kinetics were monitored by means of intrinsic fluorescence of the protein’s Trp residues, fluorescent base analogue 2-aminopurine (2aPu), and a dye–quencher pair (FAM/BHQ1). For all the fluorescent labels, the fluorescent traces showed several phases of consistent conformational changes, which were assigned to specific steps of the enzymatic process. These data offer an overall picture of the structural dynamics of AlkB and DNA during their interaction.

## 1. Introduction

**Figure 1:**
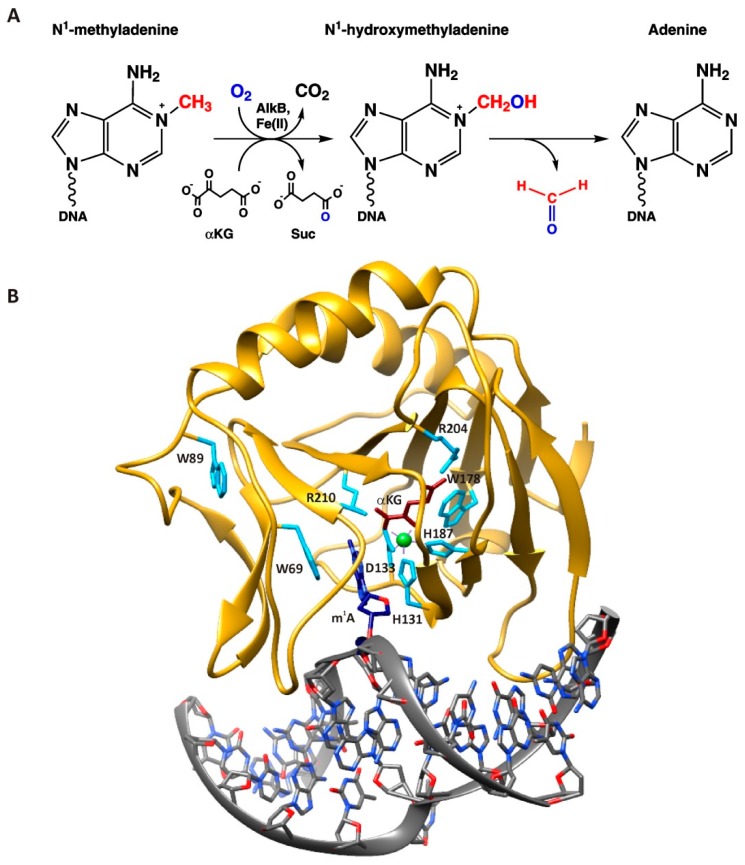
(**A**) Simplified mechanism of N^1^-methyladenine oxidative demethylation by AlkB. (**B**) Structure of AlkB complexed with dsDNA according to PDB file 3BI3 [13]. The protein and DNA backbones are yellow and grey, respectively. The m^1^A base is navy blue, αKG brown, and the Mn ion green. Amino acid residues making specific contacts with αKG, with the metal ion and with the damaged base as cyan. It should be mentioned that the crystal structure was made with the truncated form AlkB-ΔN11, so the Trp-11 residue is absent. The visualization was prepared in Chimera 1.12 [14].

Alkylated bases arise in DNA as a result of natural biochemical processes and under the influence of environmental or therapeutic agents. Accumulation of alkylated bases in the genome results in mutations and cell death. To maintain genome integrity, all organisms have evolved a variety of repair enzymes participating in distinct alkylation repair pathways. Particularly, alkylated bases are removed by AlkB family dioxygenases in direct oxidative repair [1]. The *E. coli* AlkB dioxygenase uses non-heme mononuclear iron (II) as a cofactor as well as molecular oxygen (O_2_) and α-ketoglutarate (αKG) as co-substrates, thus performing direct reversal DNA repair [2]. AlkB has been reported to remove methyl adducts (among others) from N1 atoms of adenine and guanine and from N3 atoms of cytosine and thymine in both single-stranded DNA (ssDNA) and double-stranded DNA (dsDNA) as well as ssRNA [3,4,5,6,7,8] (Figure 1A).

Crystal structures and NMR studies have revealed that similar to other dioxygenases [9,10], the AlkB protein contains a conserved double-stranded β-helix fold (“jellyroll” fold), which forms the metal- and cofactor-binding pocket [11,12]. The octahedral geometry of the primary coordination sphere around the Fe(II) atom is formed by the side chains of His-131, Asp-133, and His-187 together with O_2_ (or H_2_O under anaerobic conditions) and αKG as a bidentate ligand (Figure 1B). The αKG co-substrate is held in place by two salt bridge interactions of the carboxylate groups of αKG with Arg-204 and Arg-210. The central alkylated base within the AlkB repair complex is flipped out of the DNA helix and sandwiched between conserved residues Trp-69 and His-131, whereas the DNA backbone is distorted such that the normal bases flanking the m^1^A base stack to each other [13]. The binding of αKG and DNA blocks solvent access to the active site. Therefore, it has been suggested that O_2_ enters the catalytic center via an alternative route: Through a 3-Å-wide O_2_ diffusion tunnel [11]. This tunnel connects the oxygen-binding site with the protein surface, where the side chain of Trp-178 acts as a ‘cut-off plate’.

The key feature of the AlkB mechanism is specific conformational transitions of the enzyme molecule (including the transition from an ‘open’ to ‘closed’ conformation), which simultaneously control both the proper order of substrate binding and the kinetics of formation of a reactive oxyferryl intermediate, Fe(IV) = O [15]. It is believed that the binding of the alkylated DNA to the AlkB–Fe(II)–αKG complex initiates the following sequence of steps: (1) Displacement of H_2_O in the octahedral coordination sphere of Fe(II) by a O_2_ molecule; (2) oxidation of Fe(II) and αKG resulting in the formation of a highly reactive oxyferryl intermediate and succinate with a release of free CO_2_; (3) abstraction of the hydrogen atom from alkyl moiety in the nucleobase; and (4) subsequent spontaneous detachment of the hydroxylated alkyl group in the aldehyde form, with regeneration of the undamaged base.

Because the conformational dynamics play an important role in the catalysis by the AlkB dioxygenase [15,16,17,18], it would be interesting to correlate specific conformational transitions of the enzyme–substrate complex with the individual steps of the process. Earlier, we applied the stopped-flow (SF) kinetic analysis of conformational dynamics to several repair DNA glycosylases and apurinic/apyrimidinic endonucleases as well as their DNA substrates to demonstrate how conformational rearrangements induce and follow the recognition, binding, and chemical events [19,20,21,22]. Here, we employed the same approach to better understand the nature of the consecutive steps of the AlkB mechanism initiated by the binding of DNA substrates to the enzyme.

## 2. Results

In this work, the SF method combined with fluorescence monitoring was applied to study the conformational dynamics of AlkB. Observation of conformational changes in the enzyme–substrate complex by this pre-steady-state kinetic approach makes it possible to assign fluorescence changes to specific steps of the enzymatic reaction pathway, including substrate recognition, binding, and chemical transformation. Conformational transitions of the AlkB molecule during interactions with methylated DNA were recorded as changes in the intrinsic fluorescence of the enzyme’s Trp residues (Trp-11, Trp-69, Trp-89, and/or Trp-178). As follows from the crystal structure and NMR analysis, Trp-69 is located in a so-called ‘nucleotide-recognition lid’ (NRL) and can be involved in stacking interactions with the methylated nucleic acid base [11,15]. The side chain of Trp-89 is located on the interface of the dioxygenase core near the active site. Active-site residue Trp-178 is in direct interaction with the Fe(II) cofactor and is located on the surface of the wall of the putative O_2_-diffusion tunnel [12,23]. It has been shown that AlkB fluorescence is quenched by the addition of αKG, Suc, or methylated DNA [15]. Accordingly, we expected that changes in the local environment around these Trp residues would be sufficient for detection of the enzyme’s conformational transitions. Conformational dynamics of damaged DNA during the interactions with the AlkB dioxygenase were studied by two fluorescent probes. Firstly, to probe conformational transitions of DNA substrates in the immediate vicinity of the active site, a 2-aminopurine (2aPu) fluorescent base was introduced into the substrate sequence 3′ to N^1^-methyladenine (Table 1). In the second place, to determine the overall dynamics of the DNA structure, an m^1^A-containing oligodeoxynucleotide (ODN) was modified by the fluorescence emitter/quencher pair, FAM/BHQ1, and Förster resonance energy transfer (FRET) was measured.

### 2.1. Repair of ssDNA and dsDNA Substrates Containing an m^1^A Lesion

**Figure 2:**
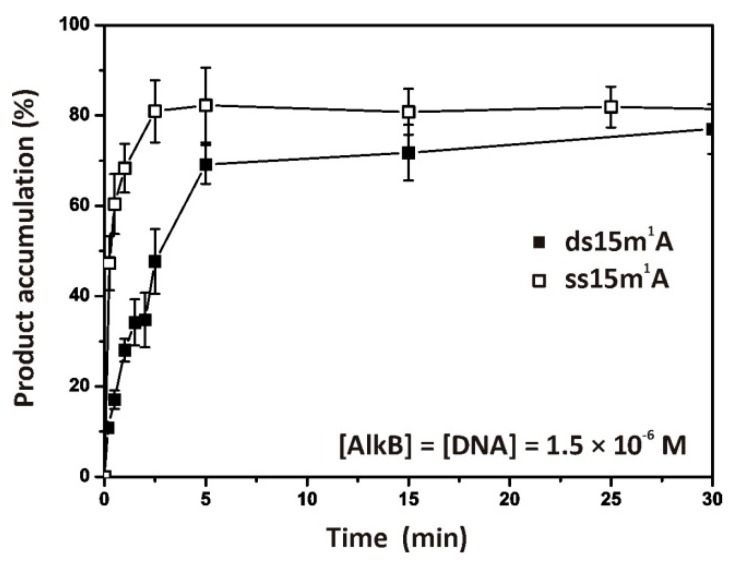
AlkB-catalyzed demethylation of ssDNA and dsDNA substrates. Time courses of AlkB-mediated product accumulation—at 37 °C followed by cleavage by DpnII (1 h, 37 °C)—were determined by denaturing PAGE analysis (autoradiograms are presented in Supplementary Materials). The experiments were conducted with equimolar amounts (1.5 μM) of AlkB and a ^32^P-labelled substrate containing the damage (m^1^A).

Firstly, the in vitro repair activity of the purified AlkB towards substrate ss15m^1^A or ds15m^1^A was studied. The m^1^A was placed next to the cleavage site of restriction enzyme DpnII to detect the repaired and cleaved ODNs by PAGE analysis (autoradiograms presented in Figure S1). Judging by the time courses of product accumulation, the degree of demethylation reaches maximal values (70%–80%) within 5 min of the interaction (Figure 2). Using Equation (1) (see the materials and methods’ section), we roughly estimated the rate of demethylation as 0.8 to 1.0 s^−1^ for the ss15m^1^A substrate and 0.5 to 0.8 s^−1^ for ds15m^1^A in terms of the *k_cat_*^PAGE^ constant. Taking into account the low accuracy of the quantitative estimation, we believe that the release of methyl groups from the ssODN and dsODN proceeds at similar efficiency rates.

Given that the 2aPu-containing substrates (Table 1) lacked the specific sequence recognized by the DpnII restriction endonuclease, it was not possible to study its demethylation by PAGE analysis. Therefore, the repair activity of AlkB towards the ss15m^1^A_2aPu substrate was confirmed by matrix-assisted laser desorption ionization time-of-flight (MALDI-TOF) mass spectrometry (Figure S2 in ‘Supplementary Materials’). As expected, more than 75% of the substrate was converted into a product after 30 min of incubation with the dioxygenase.

### 2.2. Single-Turnover Kinetics of ssDNA Demethylation by AlkB

**Figure 3:**
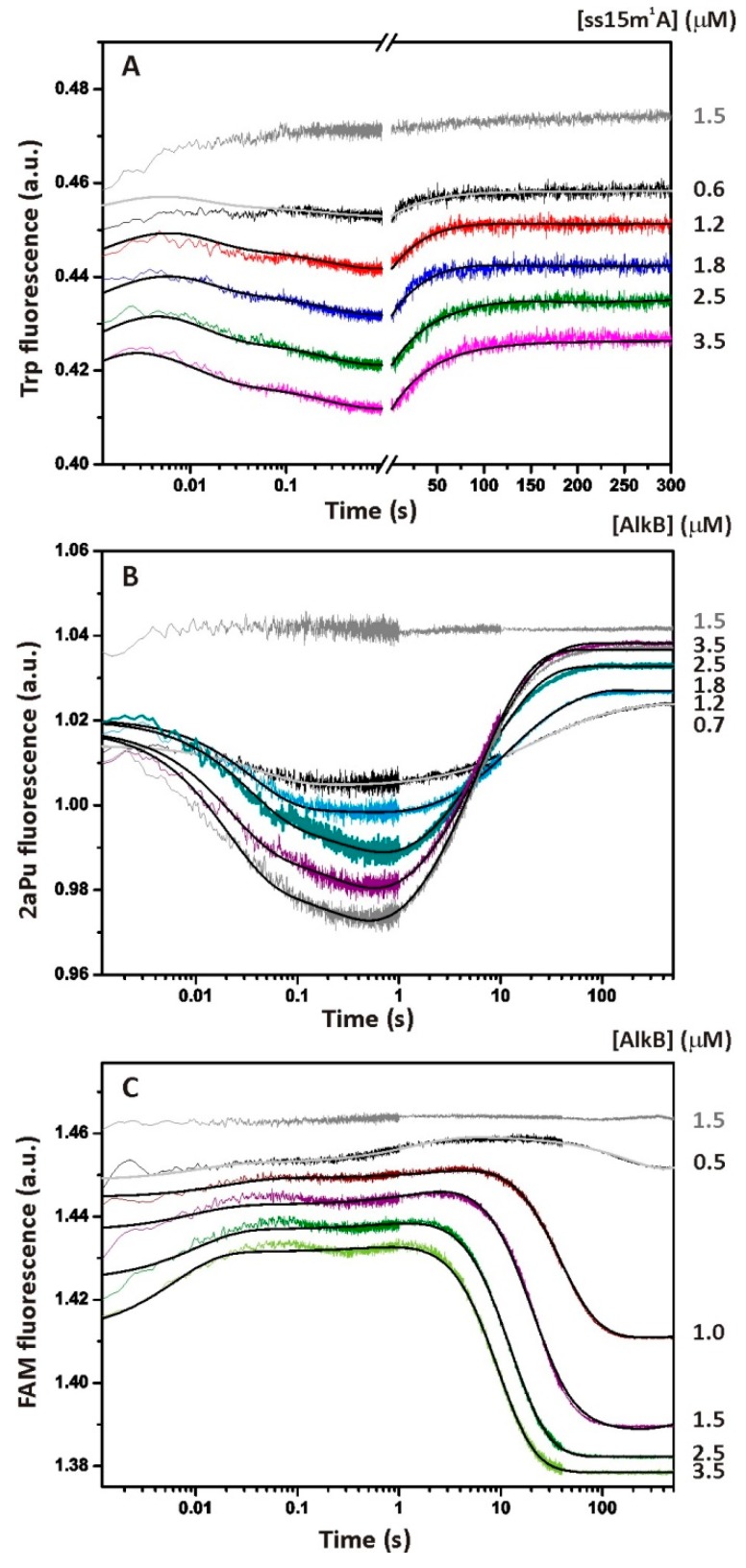
The single-turnover kinetic assay of the interaction between dioxygenase AlkB and ssDNA containing m^1^A. (**A**) Time courses of the enzyme fluorescence intensity at various concentrations of the ss15m1A substrate (0.6–3.5 μM) obtained at 37 °C. The concentration of AlkB was 1.5 μM. (**B**) Time courses of 2aPu fluorescence intensity at various concentrations of AlkB (0.7–3.5 μM). The 2aPu fluorescent base was introduced 3′ to the damage (m^1^A). The concentration of substrate ss15m^1^A_2aPu was 1.5 μM. (**C**) Time courses of the FRET signal at various concentrations of AlkB (0.5–3.5 μM). The fluorophore (FAM) and the quencher (BHQ1) were placed at the 5′ and 3′ termini, respectively. The concentration of the ss15m1A_FRET substrate was 1.5 μM. For each set of curves, jagged traces represent experimental data. Smoothed curves were obtained via global fitting of the dataset to Scheme 1. The grey curves were obtained for 1.5 μM enzyme and 1.5 μM substrate by the SF method under anaerobic conditions.

Conformational dynamics of the AlkB protein during interactions with the ss15m^1^A substrate were monitored via Trp fluorescence under single-turnover conditions. It should be noted that here and throughout by AlkB we mean the holo-protein complex AlkB–Fe(II)–αKG. The SF fluorescent traces presented in Figure 3A describe changes in the local environment of Trp residues after fast (~1 ms) mixing with damaged DNA. Structural rearrangements of this kind in most cases correspond to specific steps of an enzymatic reaction [21,24]. The resulting time courses can be divided into several phases of fluorescence growth and decay. The behavior of the initial part of the traces, expressed by the phase of the increase in the fluorescent signal between time points 1 and 5 ms, corresponds to the formation of a non-specific collisional complex. This process is controlled by diffusion. It is reported in the literature that the bimolecular diffusion rate constant, *k_diff_*, is approximately 10^8^ M^−1^s^−1^ for a protein–DNA interaction [25]. Our SF instrument can detect only the end of this process at micromolar concentrations of the reactants. The next phase of kinetic curves is accompanied by a two-step decrease in Trp fluorescence up to 1 s. We assumed that these two steps correspond to some specific conformational changes induced by recognition of the alkylated base and by the binding of dioxygen in the O_2_-diffusion tunnel that are followed by the formation of the reactive oxyferryl intermediate. In the course of these two steps, the m^1^A base should be accommodated within the active-site cavity while the octahedral coordination sphere of Fe(II) has to achieve the conformation optimal for substrate oxidation. In the next phase (1–100 s), the fluorescence grows with the amplitude, dependent on the substrate concentration, and remains constant after 100 s until the end of the registration period. After comparing the time courses of product accumulation obtained by PAGE analysis (Figure 2, empty square) with SF data (Figure 3A), we concluded that the last phase of the Trp fluorescence increase corresponds roughly to the step of substrate hydroxylation and a subsequent release of the intact DNA from AlkB.

To prove the specific nature of the observed fluorescence changes, control experiments were conducted on the SF apparatus. No changes in the enzyme fluorescence were detected if AlkB was rapidly mixed with the buffer without a DNA substrate (data not shown). We also did not detect any specific changes in the Trp fluorescence when AlkB interacted with non-methylated single- or double-stranded 15A substrate (Figure S3 in Supplementary Materials). Because an oxygen molecule is absolutely required for the catalysis by AlkB, there should be no specific changes in the enzyme conformation under anaerobic conditions. Indeed, the SF kinetic traces obtained for anaerobically prepared AlkB and the methylated ssODN uncovered changes that are close to the noise amplitude (Figure 3A–C, grey traces). Therefore, the conformational dynamics detected by the SF method under aerobic conditions reflect the specific enzyme–substrate interactions in the course of the catalytic cycle.

**Scheme 1:**
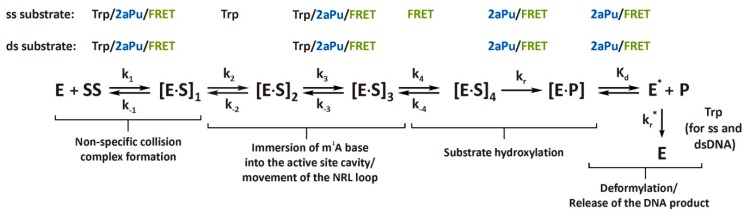
The proposed kinetic mechanism of DNA demethylation by AlkB according to the SF fluorescence data (Figure 3 and Figure 4). E is the AlkB–Fe(II)–αKG complex; E^*^ is the enzyme conformation different from E; S is a DNA substrate containing m^1^A; [E∙S]_i_ is an intermediate enzyme–substrate complex. Rate constants *k_i_* and *k_−i_*(where i = 1 to 4) characterize forward and reverse directions of the equilibria corresponding to the steps of substrate binding and adaptation for catalysis. Rate constant *k_r_* describes the irreversible step of substrate hydroxylation; *K_d_* is an equilibrium constant of the enzyme–product complex [E∙P] dissociation calculated from the DNA dynamics. The slow conformational change of AlkB at the end of the process is described by the rate constant, k_r_*. The fluorescent probes by which the individual steps were detected are highlighted above the reaction steps. A brief description of the probable processes accompanying each transient state is given under the scheme.

To study the structural dynamics of the enzyme–substrate complex in the vicinity of methylated DNA, we then recorded the DNA conformational changes by means of 2aPu fluorescence. The set of kinetic curves obtained after rapid mixing of the 2aPu-labelled DNA with AlkB revealed the conformational flexibility of the single-stranded 15m^1^A_2aPu substrate (Figure 3B). Taking into account the photophysical properties of the 2aPu residue [26], we can assume that the first phase of the signal quenching (1–500 ms) corresponds to relocation of the fluorophore into a hydrophobic environment. This process is described by two reversible steps of the kinetic mechanism (Scheme 1) and can accompany the recognition of m^1^A by AlkB as well as the stacking interactions between bases flanking m^1^A after its eversion into the active site of the enzyme [11]. The next detected phase, characterized by a sharp increase in the 2aPu signal between time points 0.6 and 50 s, may reflect the step of substrate oxidation and release of the product. This assumption follows from the fact that the fluorescence intensity of 2aPu grows with increasing hydrophilicity of the environment. Conformational changes detected by Trp and 2aPu fluorescence are generally consistent on the time scale. The difference is that the first phase of the collisional complex formation is not detected accurately by the monitoring of ss15m^1^A substrate dynamics when 2aPu is located close to the specific site. In other words, the primary binding of AlkB to the methylated ssODN has little effect on the DNA structure in the vicinity of the methylated nucleotide, whereas subsequent changes of the substrate conformation are significant.

The single-turnover kinetic curves corresponding to the FRET from 5′-FAM to 3′-BHQ1 have a complex multistep character (Figure 3C). Apparently, formation of the catalytically competent complex takes up three steps of a slight fluorescence growth and decay taking place between 1 ms and 1 s. These fluctuations coincide in a time scale with conformational transitions of the enzyme molecule detected by Trp fluorescence (Figure 3A) and most likely describe the same processes. It is known that an increase in the FRET signal is indicative of an increase in the distance between FAM and BHQ1 and vice versa. Therefore, the initial binding of the ss15m^1^A_FRET substrate to AlkB is accompanied by mutual distancing of the FRET labels. In the next phase, a slight decrease followed by a slight increase in the distance are observed, most likely reflecting the conformational transitions responsible for the formation of the catalytically competent enzyme state. The last step is expressed in a high-amplitude decrease in the FRET signal and coincides with the growth of 2aPu fluorescence in terms of the time scale (Figure 3B,C). Given that the ssDNA structure is rather flexible, we hypothesized that the termini of the 15m^1^A_FRET substrate are brought together when the ODN is free in solution. In this situation, the weakest FRET signal is detected. In the course of binding and repair by AlkB, the DNA strand adopts the conformation dictated by the enzyme active site, resulting in an increase of the FRET signal. The above explanation finds support in a comparison of the FAM fluorescence intensity of ss- and ds-substrates in solution with no enzyme added. The maximum value of fluorescence detected at 525 nm for substrate ss15m^1^A_FRET is three-fold lower than that of substrate ds15m^1^A_FRET (see Figure S4 in Supplementary Materials). Accordingly, the fluorescence of 5′-FAM is quenched by 3′-BHQ1 to a greater extent when these labels are located in the ssODN.

To assign the kinetic mechanism and individual rate constants, each set of SF data was globally fitted in DynaFit 4 software as described in our previous studies [21,22]. The summarized kinetic scheme describing the interactions of AlkB and ssDNA with sufficient accuracy (see Figure S5 for residuals) includes six steps (Scheme 1). Readers can see that not all transient states of the enzyme–substrate complex are detected by each fluorescent probe (Trp, 2aPu, or FRET), as indicated above each step of the scheme. In particular, the second conformational transition of the [E∙S]_1_ complex detected by Trp fluorescence after the initial binding of AlkB to the substrate is not detected by 2aPu and FAM fluorescence (see Figure 3). It is also apparent from the values of rate constants *k_2_^Trp^* and *k_3_^2aPu^*, which differ by a factor of 20, that these steps are related to distinct conformational transitions (Table 2). The third step of the mechanism leading to the formation of the [E∙SS]_3_ complex was detected via both the enzyme and DNA conformational dynamics and was described by the rate constant of the direct reaction of approximately 2.5 to 4.3 s^−1^. This step proceeds ~5- to 10-fold faster than the catalytic step detected by 2aPu and FRET (see *k_r_* in Table 2). Step 3 together with step 4 detected by FRET (*k_4_^FRET^* = 0.43 s^−1^) may describe conformational changes of the enzyme–substrate complex leading to the formation of the catalytically active intermediate [12] that is irreversibly hydroxylated at step 5. Using forward and reverse rate constants of steps 1–3 (Trp fluorescence) we evaluated the total affinity of AlkB for the ss15m^1^A substrate (*K_a_*) by means of the formula *K_a_* = *K_1_*+ *K_1_*× *K_2_*+ *K_1_*× *K_2_*× *K_3_*, where *K_i_* = *k_i_*/*k_−i_*. Our data indicate that *K_a_^ss^* is 2.3 × 10^8^ M^−1^, which corresponds to dissociation constant *K_d_^ss^* = 4.3 nM. The comparable affinity of the AlkB–αKG–Fe(II) complex for an m^1^A-containing trinucleotide (*K_d_* = 9.8 nM) has been reported by B. Bleijlevens et al. [16].

In terms of DNA substrate dynamics, the repair process is completed with the pronounced increase in 2aPu fluorescence and decrease of the FRET signal between 1 and 50 s. However, conformation of the protein is changing until 150 s. The *k_r_* values calculated from 2aPu fluorescence (0.47 s^−1^) or FRET (0.19 s^−1^) is much higher than that calculated from Trp fluorescence because the last slow step is characterized by rate constant k_r_* = 0.024 s^−1^ (Table 2). This value is far from the values of *k_r_^2aPu^*, *k_r_^FRET^*, and *k_cat_^PAGE^* for ss-substrates; these constants most likely represent the release of repaired DNA from the complex with AlkB. It is possible to conclude that the rate of oxidation of m^1^A should be between *k**_3_**^Trp^* and *k_r_^FRET/2aPu^*, i.e., 3.2 s^−1^ > *k_r_^ssODN^* > 0.47 s^−1^. Therefore, we suppose that after the decay of the [E∙P] complex, AlkB undergoes an additional conformational change (E* → E), which prolongs the increase in Trp fluorescence at the last step of the process (Figure 3A). This event could involve transition of the enzyme molecule to the initial ‘dynamic state’ responsible for subsequent DNA scanning for next damage [16]. Based on these findings, the last step describing the AlkB conformational transitions (Trp fluorescence) was described by general constant k_r_* (see Scheme 1 and Table 2).

### 2.3. Single-Turnover Kinetics of dsDNA Demethylation by AlkB

**Figure 4:**
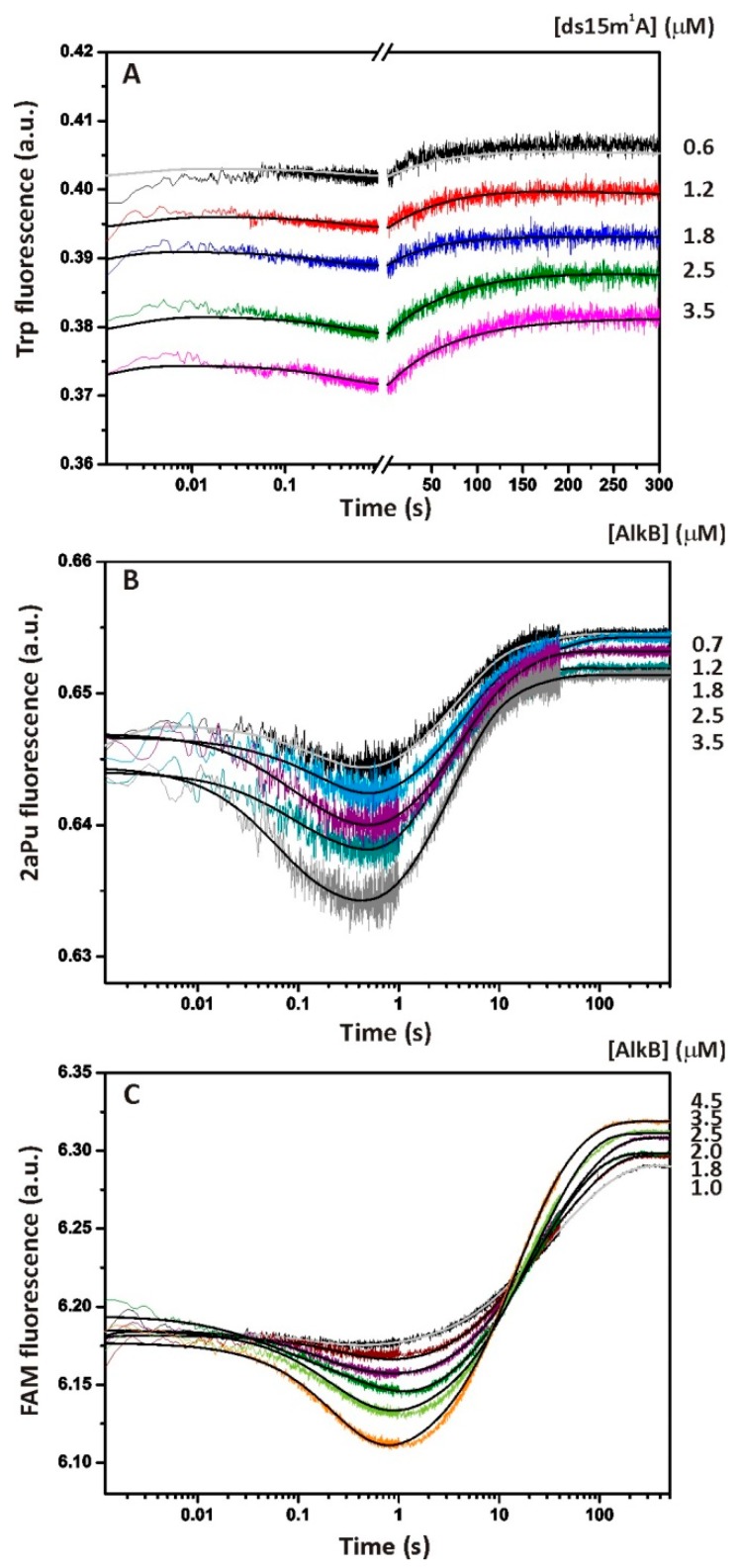
The single-turnover kinetic assay of the interaction between dioxygenase AlkB and dsDNA containing m^1^A. (**A**) Time courses of the enzyme fluorescence intensity changes at various concentrations of the ds15m^1^A substrate (0.6–3.5 μM) at 37 °C. Concentration of AlkB was 1.5 μM. (**B**) Time courses of 2aPu fluorescence intensity changes at various concentrations of AlkB (0.7–3.5 μM). The 2aPu fluorescent base was introduced 3′ to the damage (m^1^A). Concentration of substrate ds15m^1^A_2aPu was 1.5 μM. (**C**) Time courses of the FRET signal at various concentrations of AlkB (1.0–4.5 μM). The fluorophore (FAM) and quencher (BHQ1) were placed at 5′ and 3′ termini of the damaged ODN, respectively. Concentration of the ds15m^1^A_FRET substrate was 1.5 μM. For each set of curves, jagged traces represent experimental data. Smoothed curves were obtained by global fitting of the dataset to kinetic Scheme 1.

Conformational transitions during the interactions of AlkB with the ds15m^1^A substrate were studied via Trp and 2aPu fluorescence and FRET. The series of kinetic curves obtained by the SF method under single-turnover conditions are depicted in Figure 4. The shape and amplitude of the Trp fluorescent traces suggest that the conformational mobility of AlkB complexed with a dsODN is lower as compared to an ssODN, especially at the pre-catalytic steps (compare Figure 3A and Figure 4A). The quantitative analysis demonstrated that the six-step mechanism proposed for ssDNA repair is redundant in the case of the dsDNA substrate. The complexes [E∙S]_2_ and [E∙S]_4_ were kinetically indiscernible in this case, and the process was described by four steps (Scheme 1). The initial binding step is expressed as a slight increase in Trp fluorescence between 1 and 10 ms. The results of global fitting indicate that the rate constant, *k_1_*, for the dsODN (Table 2) is 2.5-fold lower than *k_1_* for the ssODN (Table 2). In addition, association constant *K_1_* (defined as the ratio *k_1_*/*k_−1_*) is one order of magnitude higher for the AlkB–ssODN complex, indicating tighter binding with the ssODN. This is probably due to increased rigidity of the duplex structure, making the compression of the nucleotides flanking the m^1^A base difficult [13]. At the second step, the signal is quenched somewhat up to 2 s, pointing to the formation of catalytically active complex [E∙S]_3_ (Scheme 1). The following growth of the enzyme fluorescence from 2 to 120 s leads to an accumulation of enzyme–product complex [E∙P]. Global analysis of the data obtained via Trp fluorescence supports the observation that the rate of ds15m^1^A (k_r_*^, dsODN^ = 0.014 s^−1^) substrate processing is a little lower than that of ss15m^1^A (k_r_*^, ssODN^ = 0.024 s^−1^; Table 2). Therefore, it can be assumed that as in the case of the ssODN, for dsODN, the slow conformational change of AlkB in the last phase of the process contributes to a lower value of k_r_*.

Conformational dynamics of the dsDNA during interactions with the AlkB dioxygenase were recorded as changes in the 2aPu fluorescence and FRET signal. As illustrated in Figure 4B, there are no specific conformational transitions detected by 2aPu fluorescence at the time points earlier than 10 ms. This means that formation of the primary [E∙S]_1_ complex does not require substantial changes in ds15m^1^A_2aPu substrate conformation. In accordance with this observation, the rate constant describing the first step of Scheme 1 via 2aPu fluorescence (*k_1_^2aPu^* = 6.6 × 10^6^ M^−1^s^−1^) is more than one order of magnitude lower than the *k_1_* value resulting from the Trp fluorescence measurements (*k_1_^Trp^* = 180 × 10^6^ M^−1^s^−1^; see Table 2). A rather different behavior of 2aPu fluorescence was observed at the second step of the enzyme–substrate complex isomerization, which was accompanied by a pronounced decrease in the signal. Once the demethylated product is formed, the 2aPu base moves away from the active site into a hydrophilic environment, as reflected by a pronounced increase in the fluorescence (Figure 4B). *k_r_^2aPu^*(ds15m^1^A) is much higher than k_r_*^, dsODN^, in agreement with the pattern observed for the ss15m^1^A substrate. Because the fluorescence of 2aPu is extremely sensitive to the polarity of the microenvironment, the increase in the 2aPu fluorescence signal reveals relocation of this fluorophore into the hydrophilic medium, meaning the release of the demethylated DNA product from the active site of AlkB. Therefore, we can hypothesize that the value of k_r_*^, dsODN^ corresponds to some post-catalytic transformation of AlkB, and the catalytic step is described by the rate constant close to or higher than *k_r_^2aPu^*. For the dsODN, the release of the intact DNA from the complex with AlkB proceeds at the rate constant of 0.45 s^−1^, which is close to the estimated values of *k_cat_^PAGE^* (0.5–0.8 s^−1^). Therefore, the real rate constant for oxidation of the methylated base within dsODN should be in the range ~0.59 s^−1^ < *k_r_^dsODN^* < 2.9 s^−1^.

Similar to the 2aPu-containing substrate, the primary binding of AlkB to substrate ds15m^1^A_FRET did not yield notable changes in the FAM-BHQ1 distance (up to 20 ms). Eversion of the m^1^A base and interaction of the NRL amino acid residues with the duplex stack at the second step lead to quenching of the FAM fluorescence in the interval from 50 to 800 ms. (Figure 4C). The reduction in the distance between the DNA termini causes bending of the double helix, thereby facilitating the flipping-out of the m^1^A base. The described conformational change matches the phases of 2aPu and Trp fluorescence quenching; consequently, we can propose that the third step of Scheme 1 represents an important conformational transition that affects each participant of the catalytic complex. The fourth step, corresponding to a substantial increase in the FRET signal, is irreversible oxidation of the methylated substrate and is described by the rate constant *k_r_^FRET^* of 0.21 s^−1^.

### 2.4. Interaction of AlkB with Undamaged DNA Representing the Reaction Product

**Figure 5:**
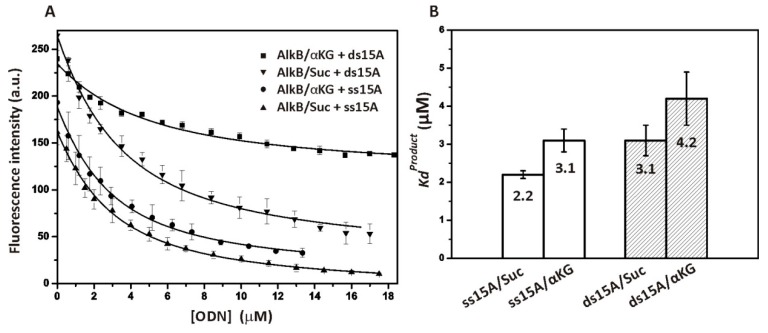
Intrinsic Trp fluorescence assays of undamaged DNA binding to AlkB. (**A**) The protein at 1.5 μM was titrated with an undamaged ssODN (ss15A) or dsODN (ds15A) in the presence of the Fe(II) cofactor (40 μM) and co-substrate αKG (1 mM) or co-product Suc (2 mM). (**B**) The values of the dissociation constant *K_d_^Product^* were obtained by fitting the data to a one-step binding scheme. Error bars denote the standard error of three iterations.

To determine the affinity of the AlkB protein for the demethylated product, equilibrium fluorescent titration was performed. The enzyme solution contained the Fe(II) cofactor (40 µM) and co-substrate αKG (1 mM) or co-product Suc (2 mM). Judging by Figure 5A, Trp fluorescence is quenched by the addition of an undamaged ssODN or dsODN (ss15A or ds15A, see Table 1). The data were fitted to the one-step binding mechanism, E + P ⇄ EP, and the equilibrium dissociation constant *K_d_^Product^* was determined (Figure 5B). The values of *K_d_^Product^* lie in the micromolar range, indicating the moderate affinity of AlkB for intact DNA. Our data suggest that AlkB forms the best complex with the ss15A ODN in the presence of co-product Suc and the weakest one with the ds15A ODN in the presence of co-substrate αKG. These findings are consistent with the values of the dissociation constant *K_d_* obtained by the global fitting of SF data (see Table 2). A similar binding affinity of an undamaged 5 nt ODN for the AlkB–Suc complex (~0.9 μM) at 25 °C has been reported in another study [16].

## 3. Discussion

AlkB is a DNA repair enzyme firstly discovered in 1977 [27] as a component of the ‘adaptive response’ in bacterial cells. Using the sequence homology alignment, Aravind and Koonin predicted in 2001 that AlkB is a Fe(II)/α-ketoglutarate-dependent dioxygenase [2]. The enzymes of the AlkB family are widely distributed throughout the kingdoms of life [28]. The general knowledge about the catalytic mechanism of non-heme dioxygenases is constantly expanding with new results. Multiple kinetic and spectroscopic studies have evaluated the rate of the formation and decay of the Fe(IV)−oxo intermediate [29,30,31,32] and the efficiency of the binding of co-substrate αKG, co-product Suc, and metal ion to the enzyme [12,15,33]. Some authors have shown the importance of protein dynamics for efficient co-substrate binding and catalysis [16,17]. However, there is little information about the conformational dynamics of the enzyme–substrate complex in the course of demethylation.

**Figure 6:**
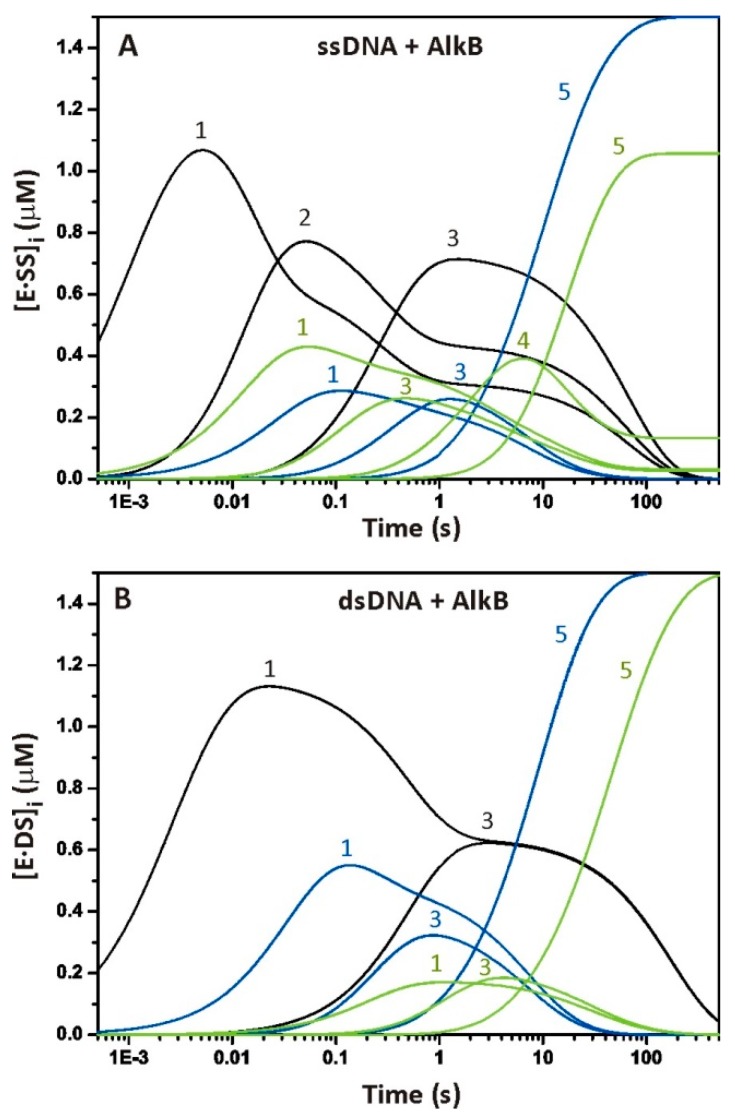
Kinetic simulation of the time courses of the appearance and disappearance of various intermediates according to SF data. (**A**) Transient complexes between AlkB and single-stranded substrates [E∙S]_i_ according to Trp fluorescence (black curve), 2aPu fluorescence (blue line), and the FRET signal (green curve). (**B**) Transient complexes between AlkB and ds-substrates [E∙S]_i_ according to Trp fluorescence (black curve), 2aPu fluorescence (blue curve), and the FRET signal (green curve). The simulations were run at [E]_0_ = [S]_0_ = 1.5 μM. The intermediates are labelled according to the numbers of transient complexes in the Scheme 1. The intermediate 5 describes the formation of the enzyme-product complex [EP].

Here, we applied the pre-steady-state kinetic approach to describe individual conformational states of the AlkB–DNA complex during the pre-steady-state phase of the process. The findings suggest that conformational fluctuations are an inherent property of AlkB catalysis. We found that the enzyme–substrate complex with ssDNA is more flexible and undergoes more conformational transitions than that of dsDNA. To analyze the behavior of the transient complexes detected by means of Trp and 2aPu fluorescence and FRET, we simulated the reaction pathway with the rate constants from Scheme 1 for ss- and ds-substrates. Figure 6 presents time courses of the formation and consumption of intermediate species. The first step in these schemes leads to the formation of complex [E∙S]_1_^Trp^, representing the non-specific initial binding of AlkB to methylated DNA. As discussed in the results, the formation of complex [E∙S]_1_^Trp^ precedes the formation of [E∙S]_1_^2aPu^ and [E∙S]_1_^FRET^ (Figure 6A). Thus, the first phase of the formation of a collisional complex between the enzyme and its substrate is not detected by 2aPu fluorescence and FRET. In other words, the primary binding of AlkB to the methylated ssODN and dsODN does not alter the DNA structure substantially. It has been demonstrated in chemical cross-linking experiments that AlkB recognizes its substrate by sensing lesion-induced structural distortions in a DNA sequence [34]. At this point, we can propose that the flipping-out of an m^1^A base occurs at the second step of the process and is reflected by the protein and DNA dynamics (see the emergence of complexes [E∙S]_2_^Trp^, [E∙S]_1_^2aPu^, and [E∙S]_1_^FRET^ in Figure 6A). This transition is accompanied by mutual distancing of the FRET labels and by quenching of 2aPu fluorescence due to stacking interactions between G_5_ and 2aPu_7_. As follows from the simulation diagram, the formation of species [E∙S]_3_^Trp^, [E∙S]_3_^2aPu^, and [E∙S]_3_^FRET^ proceeds simultaneously, whereby [E∙S]_3_^Trp^ is consumed more slowly than the others are. Additional experiments are needed to determine precisely which events or actions accompany these conformational transitions. Nevertheless, it is clear that step 3 of Scheme 1 corresponds to a global conformational change, affecting both the enzyme and DNA structure. It may include the docking of the methylated base by a flexible loop of the NRL, diffusion of dioxygen to the active site, or αKG co-substrate oxidation. The oxidation mechanism used by Fe(II)/αKG-dependent dioxygenases requires the formation of the ternary complex E-Fe(II)-αKG-substrate before activation of the O_2_ molecule. Previous studies reported the rate of oxyferryl intermediate formation from 30 s^−1^ (for taurine hydroxylase) to 1.7 s^−1^ (for DAOCS dioxygenase), and even 0.018 s^−1^ (for PHD2 dioxygenase) [31,35,36]. Therefore, under the conditions of our experiments, the active oxyferryl intermediate should arise after the specific enzyme–substrate complex formation but before the substrate hydroxylation. The rate of this process may lie between 0.063 and 3.5 s^−1^, and this assumption is consistent with the observation that the lifetime of the oxyferryl intermediate in the case of AlkB is ≥11 s [15].

Regarding dsDNA, the primary binding to AlkB is also detected by changes in the enzyme conformation (Figure 6B). The [E∙S]_1_^FRET^ complex forms later than [E∙S]_1_^2aPu^ does, which may arise from the reduced susceptibility of the duplex structure to bending and distortion as compared to ssDNA. Thus, the distance between the FRET labels at the substrate termini is not affected by non-specific binding and eversion of the m^1^A base. The convergence of FAM and BHQ1 that resulted from the [E∙S]_3_^FRET^ complex formation probably describes a transition of the catalytically active conformation.

The formation of complexes [E∙P]^2aPu^ and [E∙P]^FRET^ corresponds to the final catalytic steps, leading to hydroxylation of the methylated base and a subsequent spontaneous formaldehyde release with restoration of the native adenine structure because it is in line with the rate of product formation detected by PAGE analysis. The mean value of *k_r_* = 0.33 ± 0.14 s^−1^ resulting from constants *k_r_^ssODN^* (0.19–0.47 s^−1^) and *k_r_^dsODN^* (0.21–0.45 s^−1^) (observed at 37 °C) is consistent with the oxidation rate constant reported earlier (more than 0.43 s^−1^ at 10 °C) [15]. At the same time, the SF kinetics determined by Trp fluorescence detection suggest that the final enzyme complexes for the ss-substrate and ds-substrate (Figure 6A,B; black curve) emerge later than complexes [E∙P]^2aPu^ and [E∙P]^FRET^ do. Thus, the slow increase in the signal at the end of the Trp fluorescence traces most likely means post-catalytic transformation of the AlkB structure. Further studies are needed to elucidate the nature of this conformational transition. On the basis of available studies on AlkB structure-function relationships [17], we can assume that this change in the AlkB structure after the release of the DNA product is caused by a transition of the double-stranded β-helix fold to a less folded state required for the removal of the Suc co-product from the AlkB–Fe(II)–Suc complex.

## 4. Materials and Methods

### 4.1. ODNs

ODNs (Table 1) containing normal DNA bases and N^1^-methyladenine (m^1^A) and/or 2-aminopurine (2aPu) and FRET labels were synthesized on an ASM-700 Synthesizer (BIOSSET Ltd., Novosibirsk, Russia) using phosphoramidites purchased from Glen Research (Sterling, VA, USA) and were purified by anion exchange high-performance liquid chromatography. Concentrations of the ODNs were determined by measuring absorbance at 260 nm (A_260_). The purity, homogeneity, and integrity of each ODN were assessed by 20% polyacrylamide gel electrophoresis (PAGE) followed by Stains-All dye staining (Sigma-Aldrich, St. Louis, MO, USA). When needed, the modified DNA strands were ^32^P-labelled using [γ-^32^P]ATP and bacteriophage T4 polynucleotide kinase (SibEnzyme, Novosibirsk, Russia) and were purified by 20% denaturing PAGE.

Double-stranded substrate duplexes were prepared by annealing the modified strands (15m^1^A, 15m^1^A_2aPu, or 15m^1^A_FRET) and complementary strands (15T or 15TT in the case of a 2aPu-containing substrate) in a 1:1 molar ratio in a buffer consisting of 50 mM HEPES-KOH pH 7.5, 50 mM KCl, and 10 mM MgCl_2_. Single-stranded substrates have the ‘ss’ prefix, and double-stranded substrates the ‘ds’ prefix.

### 4.2. Protein Expression and Purification

The full-length His_6_-tagged AlkB protein was isolated from *E. coli* Rosetta 2 cells transformed with plasmid pET28a carrying the wild-type *alkb* gene. The cells were grown in the Luria–Bertani medium containing 50 μg/mL kanamycin at 37 °C to A_600_ of 0.6 to 0.7. After induction with isopropyl β-d-1-thiogalactopyranoside (IPTG; 0.2 mM), the cell culture was incubated at room temperature overnight. The cells were lysed in a French pressure cell press (SLM Aminco). All the subsequent procedures were conducted at 4 °C. The cell lysate was centrifuged (30,000× *g*, 40 min), and the supernatant was loaded onto a Q-Sepharose Fast Flow column (GE Healthcare Bio-Sciences, Pittsburgh, PA, USA) with subsequent washing with a buffer consisting of 20 mM HEPES-KOH (pH 7.8) and 40 mM NaCl. Fractions containing the protein [37] were collected and loaded onto a HiTrap Chelating HP nickel column (Amersham Biosciences) in a buffer consisting of 20 mM HEPES-KOH (pH 7.8), 500 mM NaCl, and 10 mM imidazole. The chromatography was run in the same buffer with a linear 10–500 mM gradient of imidazole. The fraction containing the AlkB protein was dialyzed against a buffer (20 mM Tris-HCl pH 8.0, 100 mM NaCl, 1 mM DTT, and 50% of glycerol) and stored at −20 °C. The homogeneity of the protein was verified by SDS-PAGE (Figure S6 in Supplementary Materials). The concentration of the protein was determined by the Bradford assay.

### 4.3. A DNA Repair Assay

The repair activity assay was performed on the purified AlkB protein by incubation of 1.5 μM enzyme with 1.5 μM [^32^P]-labelled ssDNA or dsDNA substrate at 37 °C in reaction buffer (50 mM HEPES-KOH pH 7.5, 50 mM KCl, 10 mM MgCl_2_, 1 mM αKG, 2 mM Na ascorbate, and 40 μM (NH_4_)_2_Fe(SO_4_)_2_·6H_2_O). To terminate the reaction at each time point, 5-μL aliquots were added to an equal volume of 0.2 M NaOH. Aliquots were then desalted on a MicroSpin G-25 column (GE Healthcare) and diluted by the restriction buffer for DpnII (New England Biolabs, Inc.). Digestion with the DpnII restriction enzyme was carried out at 37 °C for 60 min as described previously [37]. In the case of the ss15m^1^A substrate, a two-fold molar excess of the complementary ODN (15T) was added to aliquots prior to the restriction digestion. The reaction products were separated by 20% denaturing PAGE. Each experiment was repeated three times. The radioactivity was detected by autoradiography of the gel and quantified by scanning densitometry in Gel-Pro Analyzer 4.0 software (Media Cybernetics, Silver Spring, MD). The data were analyzed under the assumption that a catalytic rate constant is a ratio of the initial velocity, *V_0_*, to the equilibrium concentration of the pre-catalytic complex [E∙SS]_4_ (or [E∙DS]_2_) using the following equation:

(1) $$k_{cat}^{PAGE}=2V_{0}{\left(e_{0}+s_{0}+\frac{1}{K_{a}}+\sqrt{{\left(e_{0}+s_{0}+\frac{1}{K_{a}}\right)}^{2}-4e_{0}s_{0}}\right)}^{-1}$$

where *V_0_* is the initial velocity calculated from the slope of the linear part of the PAGE time course; *e_0_* and *s_0_* are the total concentrations of the enzyme and DNA; and *K_a_* is an equilibrium association constant computed from the values of direct and reverse rate constants of the steps determined in the quantitative analysis of SF data: *K_a_* = *K_1_*+ *K_1_*× *K_2_*+ *K_1_*× *K_2_*× *K_3_*, where *K_i_* = *k_i_*/*k_−i_*, and *i* is an ID number of a step.

### 4.4. SF Fluorescence Measurements

These measurements were carried out using a model SX.20 stopped-flow spectrometer (Applied Photophysics, Leatherhead, UK) equipped with a 150 W Xe arc lamp. The fluorescence of Trp was excited at λ_ex_ = 300 nm and monitored at λ_em_ > 320 nm via a long pass filter WG-320 (Schott, Mainz, Germany). The 2aPu fluorescent probe was excited at 310 nm and detected at >370 nm (LG-370 filter, Corion, Franklin, MA, USA). To detect the signal of Förster resonance energy transfer (FRET), the fluorescence of the FAM label was excited at 494 nm. The emission was registered at wavelengths >530 nm using the OG-530 filter (Schott). All the kinetic curves were recorded under aerobic conditions, i.e., all the aqueous solutions contained 2 × 10^−4^ M O_2_. The SF syringes were loaded with solutions of a DNA substrate and enzyme in the standard reaction buffer described in Section 4.3. After fast mixing of the reagents in a reaction chamber (dead time of the instrument is 1 ms), a time course of fluorescence changes was determined at 37 °C. Typically, each trace shown is the average of at least five independent trials. Because the fluorescence changes were detected in a wide range of time, each trace was registered in split mode, including the following intervals: 1 ms to 1 s, 1 to 40 s, and 1 to 500 s. The concentration of AlkB was 1.5 μM when Trp fluorescence was measured, and the concentration of the ODN substrate varied from 0.6 to 3.5 μM. During the detection of 2aPu or FAM fluorescence, the enzyme concentration was varied in the 0.5–4.5 μM range, and the substrate concentration was 1.5 μM. To construct the SF kinetic curves under anaerobic conditions, the reagent solutions were prepared as follows. A buffer solution (30 mL) consisting of 50 mM HEPES-KOH pH 7.5, 50 mM KCl, and 10 mM MgCl_2_ was degassed on a vacuum pump Büchi Vac V-1000 (BÜCHI Labortechnik, Flawil, Switzerland) and transferred into an inert-atmosphere box filled with argon. The buffer solution was bubbled with argon gas during 30 min and used for the preparation of the substrate and enzyme solutions hereafter. The DNA substrate (3 μM) and enzyme (3 μM) solutions containing Fe(II) (40 μM), αKG (1 mM) and ascorbate (2 mM) were prepared in the glove box separately and then bubbled with argon during 10 min on ice. The SF system was washed with anaerobic buffer solution and rapidly loaded with the enzyme and substrate. The fluorescence time courses for each fluorescent probe were recorded at 37 °C.

### 4.5. Global Fitting of SF Data

Determination of the kinetic mechanism and of the number of individual reaction steps was implemented as follows. Each fluorescence trace from one set was fitted to the sum of exponentials, and the observed rate constants, k_i_^*^, were determined:

(2) $$F=a_{1}exp\left(-k_{1}^{*}t\right)+a_{2}\left(1-exp\left(-k_{2}^{*}t\right)\right)+C$$

where *F* is the fluorescence intensity at any reaction time point *t*, *a_1_* and *a_2_* are the amplitudes, and k^*^_1_ and k^*^_2_ are the observed rate constants. Values of the observed rate constants were plotted versus DNA or enzyme concentrations to estimate the maximal observed rate and *y*-intercept. The results subsequently served as initial values in a global fitting analysis. Global non-linear least-squares fitting was performed with the DynaFit software (BioKin, Pullman, WA, USA) [38]. This approach is based on fluorescence intensity variation in the course of the reaction owing to sequential formation and further transformation of the initially formed DNA–enzyme complex and its conformers. The SF Trp fluorescence traces were directly fitted to the expression of fluorescence intensity (*F*) at any reaction time point, *t*, as the sum of the background fluorescence (*F_b_*) and fluorescence intensities of all protein species:

(3) $$F=F_{b}+\overset{n}{\underset{i=0}{\sum }}F_{i}\left(t\right)$$

(4) $$F_{i}\left(t\right)=f_{i}\times \left[E_{i}\left(t\right)\right]$$

where *f_i_* is the coefficient of the specific fluorescence for each discernible AlkB conformer, and [*E_i_(t)*] is the concentration of the conformer at any given time point *t* (*i* = 0 corresponds to the free protein, and *i* > 0 to protein–DNA complexes). These specific fluorescence coefficients describe only the part of the fluorescence that changes due to the DNA binding. The software performs numerical integration of a system of ordinary differential equations with subsequent non-linear least-squares regression analysis. Similar fitting procedures were carried out for 2aPu fluorescence and FRET traces except that fluorescently discernible transient states corresponded to DNA species. The validity of the proposed reaction schemes was confirmed by a ‘scree test’ [39]. Error in all rate constant values was determined as the standard deviation of kinetic constants derived from different fitting iterations.

### 4.6. Fluorescence Equilibrium Titration of AlkB

To determine the equilibrium dissociation constant (*K_d_^Product^*) of the complex between AlkB and an undamaged ODN (ss15A or ds15A), the Trp fluorescence intensity was measured during titration of 1.5 µM AlkB with various concentrations of an ODN in a buffer consisting of 50 mM HEPES-KOH (pH 7.5), 50 mM KCl, 10 mM MgCl_2_, 1 mM αKG, and 40 μM (NH_4_)_2_Fe(SO_4_)_2_·6H_2_O. The mixtures were incubated at 25 °C for 30 s, and fluorescence intensity was recorded (λ_ex_ = 280 nm, λ_em_ = 330 nm) on a Cary Eclipse fluorescence spectrophotometer (Agilent, Santa Clara, CA, USA). The results of three independent titrations were averaged, and the data were analyzed via Equation (5) in the OriginPro 8 software (OriginLab Corp, Northampton, MA, USA):

(5) $$F=f_{0}+f_{2}e_{0}+\frac{\left(f_{1}-f_{2}\right)}{2}\left(\sqrt{{\left(K_{d}^{Product}+p_{0}-e_{0}\right)}^{2}+4K_{d}^{Product}e_{0}}-K_{d}^{Product}-p_{0}+e_{0}\right)$$

where *F* is the Trp fluorescence intensity measured for each ODN concentration; *f_0_*, *f_1_*, and *f_2_* are the fluorescence intensities of AlkB with no ODNs added at any given ODN concentration or at the saturating ODN concentration, respectively; and *e_0_* and *p_0_* are the total concentrations of AlkB and ODNs, respectively.

## Figures and Tables

**Table 1:** Sequences of the oligodeoxynucleotides (ODNs) used in this study.

Name	Sequence ^1^
15A	5′-ACAGG**A**TCCGGCATA-3′ ^2^
15m^1^A	5′-ACAGG**m^1^A**TCCGGCATA-3′
15m^1^A_2aPu	5′-ACAGG**m^1^A(2aPu)**CCGGCATA-3′
15m^1^A_FRET	5′-FAM-ACAGG**m^1^A**TCCGGCATA-BHQ1-3′
15T	5′-TATGCCGGATCCTGT-3′
15TT	5′-TATGCCGGTTCCTGT-3′

^1^ m^1^A: N^1^-methyladenine, 2aPu: 2-aminopurine, FAM: 6-carboxyfluorescein, BHQ1: black hole quencher 1, FRET: Förster resonance energy transfer. ^2^ The underlined sequence GATC is specifically recognized by the DpnII restriction enzyme.

**Table 2:** A summary of kinetic parameters for the process of ss15m^1^A substrate demethylation by the AlkB protein, as determined by the global fitting of SF kinetic data to Scheme 1 (37 °C). The color code assigned to each fluorescent probe corresponds to the legend of Scheme 1.

Substrate	ss15m^1^A	ss15m^1^A_2aPu	ss15m^1^A_FRET	ds15m^1^A	ds15m^1^A_2aPu	ds15m^1^A_FRET
*k_1_* × *10^-6^*, *M^−1^ s^−1^*	455 ± 33	13 ± 1	19 ± 4	180 ± 80	6.6 ± 0.9	0.28 ± 0.03
*k_−1_, s^−1^*	9.2 ± 5	8 ± 0.5	51 ± 8	34 ± 13	9.2 ± 1.5	3.8 ± 0.2
*k_2_, s^−1^*	49 ± 4					
*k_-2_, s^−1^*	35 ± 3					
*k_3_, s^−1^*	3.2 ± 0.3	2.5 ± 0.4	4.3 ± 1.5	1.1 ± 0.2	2.5 ± 0.8	1.1 ± 0.2
*k_-3_, s^−1^*	1.9 ± 0.2	1.5 ± 0.1	3.8 ± 0.6	1.1 ± 0.1	2.9 ± 0.3	0.78 ± 0.07
*k_4_, s^−1^*			0.43 ± 0.12			
*k_-4_, s^−1^*			0.063 ± 0.015			
*k_r_, s^−1^*		0.47 ± 0.04	0.19 ± 0.03		0.45 ± 0.11	0.21 ± 0.04
*K_d,_ M*		(2.2 ± 0.3) × 10^−6^	(3.1 ± 0.8) × 10^−7^		(1.8 ± 0.2) × 10^−6^	(4.4 ± 0.2) × 10^−6^
k_r_*, s^−1^	0.024 ± 0.004			0.014 ± 0.001		
Fluorescent probe	Trp	2aPu	FRET	Trp	2aPu	FRET

## Supplementary Materials

The following are available online at https://www.mdpi.com/1420-3049/24/24/4576/s1, Figure S1: PAGE analysis of AlkB repair activity towards model substrates, Figure S2: MALDI-TOF mass-spectrometric analysis of the reaction product generated by the incubation of AlkB with substrate 15m^1^A_2aPu containing a 2aPu fluorescent base, Figure S3: The SF time courses of Trp fluorescence obtained under interactions of AlkB and non-methylated DNA, Figure S4: The emission spectrum of the FAM label within the ssDNA or dsDNA substrates, Figure S5: Representative graphs of the residuals for fitting of experimental data, Figure S6: SDS PAGE gel analysis of the AlkB protein purity and homogeneity.
