# Supplementary material for: A Single-Turnover Kinetic Study of DNA Demethylation Catalyzed by Fe(II)/α-Ketoglutarate-Dependent Dioxygenase AlkB
Source: Molecules. 2019 Dec 13;24(24):4576. doi: 10.3390/molecules24244576 (PMC6943663; doi:10.3390/molecules24244576)
Supplement: Supplementary file 1 [file molecules-24-04576-s001.pdf]

Supplementary Materials

Supplementary methods

MALDI-TOF mass-spectrometric analysis of the AlkB activity. The demethylation activity of AlkB towards 2aPu-containing substrates was confirmed by MALDI-TOF mass spectrometry on a Bruker REFLEX III instrument at the Joint Center for Genomic, Proteomic and Metabolomics Studies of ICBFM (Novosibirsk, Russia). The experiments were conducted with the free substrate (ss15m^{1}A_2aPu) and free product (ss15A_2aPu) in a reaction mixture consisting of 1.5 μM substrate, 15 μM AlkB, 50 mM HEPES-KOH (pH 7.5), 50 mM KCl, 10 mM $MgCl_2$, 1 mM αKG, 2 mM sodium ascorbate and 40 μM $(NH_4)_2Fe(SO_4)_2$ $6H_2O$. After incubation at 37 ℃ for 30 min, the reaction products were precipitated with 2% lithium perchlorate in acetone and desalted in a ZipTipC$_{18}$ pipette tip (Millipore, Germany). The spectra were acquired in negative mode using the 3-hydroxypicolinic matrix in 10 mM ammonium citrate.

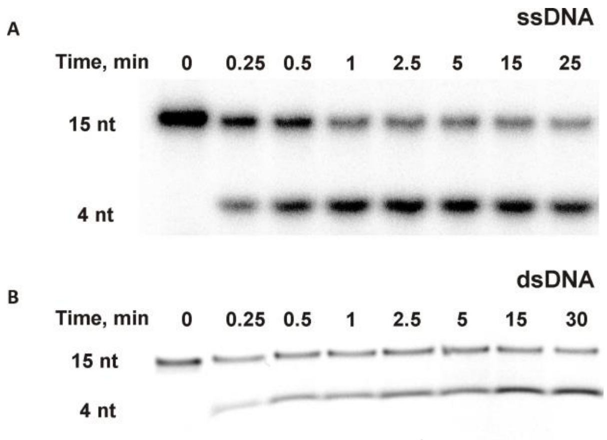

Figure S1. PAGE analysis of AlkB repair activity towards model substrates. AlkB at 1.5 μM was incubated with an equimolar amount of a ^{32}P-labelled ss~~ (or ds) ~~15m^{1}A (**A**) or ds15m^{1}A substrate (**B**). The reaction was quenched at each time point by the addition of an equal volume of 0.2 M NaOH. After thorough purification, each probe was treated with the DpnII enzyme, which is specific to non-methylated GATC motif~~s~~, and was analysed by denaturing PAGE. Each chemical quench experiment was carried out three times. Panels (**A**) and (**B**) represent the typical one.

Formatted: Font: Bold

Formatted: Font: Bold

Formatted: Font: Bold

Formatted: Font: Bold

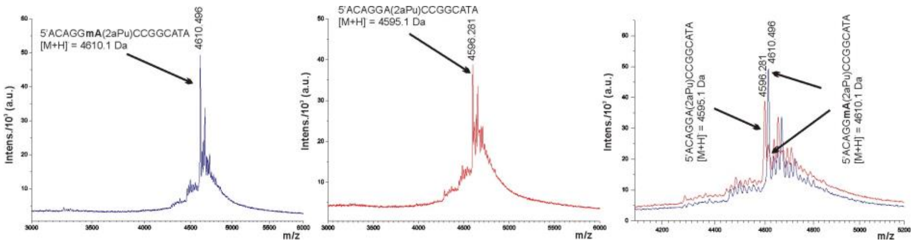

Figure S2. MALDI-TOF mass-spectrometric analysis of the reaction product generated by the incubation of AlkB with substrate 15m^{1}A_2aPu containing a 2aPu fluorescent base. Three probes were analysed by mass spectrometry in negative mode on the 3-hydroxy picolinic acid matrix: ODN 15m^{1}A_2aPu corresponding to the methylated substrate **(left panel)**, ODN 15A_2aPu corresponding to the undamaged product **(central panel)** and a reaction mixture **(right panel)**.

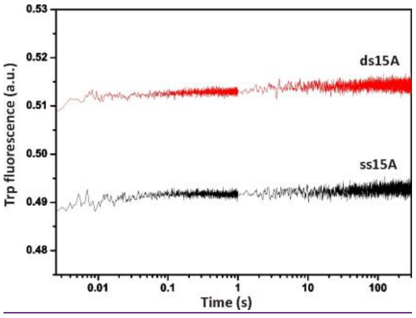

Formatted: Centered

Figure S3. The SF time courses of Trp fluorescence obtained under interactions of AlkB and non-methylated DNA. The single- and double stranded DNA substrates of 15 nt length contained the adenine residue instead of m^{1}A. Equal concentrations of the enzyme and substrate were used (1.5 µM). All experimental conditions were the similar to those of SF experiments with methylated DNA.

Formatted: Font: Bold

Formatted: Font: Bold

Formatted: Superscript

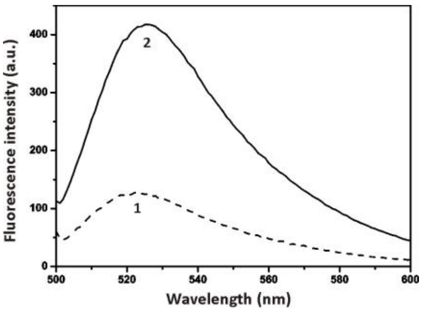

Figure ~~S3~~S4. The emission spectrum of the FAM label within the ssDNA or dsDNA substrates. *1*: The emission spectrum of substrate ss15m^{1}A_FRET. *2:* The emission spectrum of substrate ds15m^{1}A_FRET. λ_{ex} = 494 nm. The spectra were recorded in solutions consisting of 1.5 μM substrate, 50 mM HEPES-KOH (pH 7.5), 50 mM KCl and 10 mM $MgCl_2$.

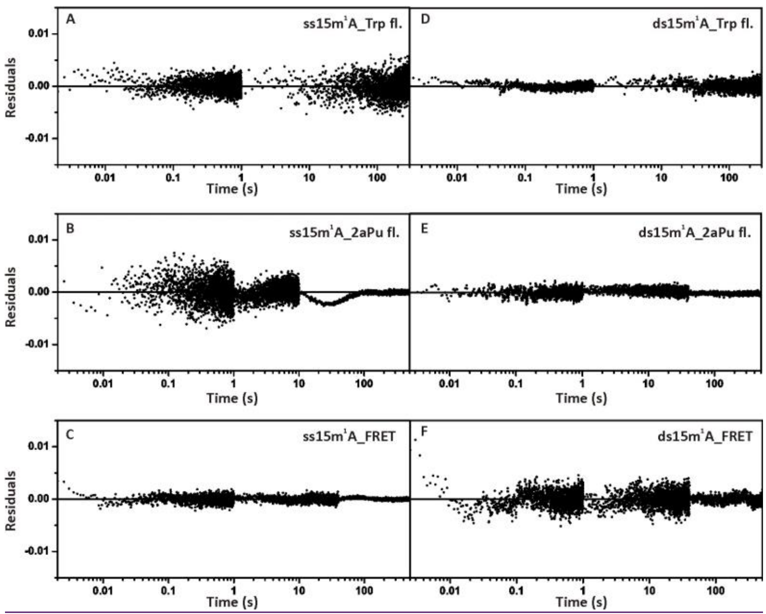

Figure S5. Representative graphs of the residuals for fitting of experimental data. Residuals, which describe the SF data obtained for ssDNA substrates (Fig. 3), are shown in panels A-C. Residuals for dsDNA substrates (Fig 4.) are shown in panels D-F.

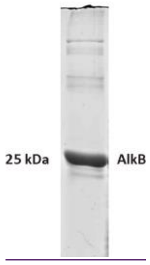

Figure S6. SDS PAGE gel analysis of the AlkB protein purity and homogeneity.

Formatted: Centered

Formatted: Font: Bold

Formatted: Justified, Line spacing: 1.5 lines

Formatted: Centered, Line spacing: 1.5 lines

Formatted: English (United States)

Formatted: Justified, Line spacing: 1.5 lines

Formatted: English (United States)

Formatted: English (United States)
